# The Dispensaries of the Punjab

**Published:** 1900-12-29

**Authors:** 


### The Institutional Workshop.

### THE DISPENSARIES OF THE PUNJAB.

Five new dispensaries were opened during the year 1898, and three were closed, for which course, however, satisfactory reasons were forthcoming. The total number now receiving patients throughout the Punjab is 267, the report of three years ago giving almost the same figures. There is still urgent need for more dispensaries, especially in Hazára, Umballa, Lahore, and Siálkot, where the number of people to a dispensary varies from 172,000 to 124,000. In fact, there is not a district in the Punjab which would not be the better for an increase in the hospitals. But ways and means have to be considered, and even if that difficulty were overcome, it would be no easy matter to find enough medical subordinates to work the dispensaries. More men, it is stated, are being trained each year, and in time dispensaries are bound to multiply, but it will be long before there are as many as are needed. There was a large decrease in the total of patients treated in 1898, 268,535 less coming forward than in 1897; but as the increase had been a substantial one in the previous year, the falling-off in numbers can hardly be due to a falling-off of appreciation on the part of the natives. It is rather due to the extreme healthiness of the year, and also to the fact that more care has been exercised to prevent the hospitals being turned into poor-houses, patients suitable for the latter no longer being received. In addition, many dispensaries had to be closed in order that the staff might be sent on plague duty, and many civil surgeons were taken away from their charges for the same purpose. Men were for unavoidable causes constantly being changed about during the greater part of the year, this naturally destroying confidence, so that it is surprising the decrease was not more marked. The number of beds available for the year was 3,095, which except in a few hospitals has been found to be considerably in excess of the total required. The authorities find it exceedingly difficult to reduce this excess with advantage, for a certain number of beds must be reserved for urgent cases; and when once a hospital is built the beds, if unoccupied, cost the hospital nothing beyond the repairs of the rooms in which they stand. An effort is being made to so arrange that other rooms than the wards might be available in case of pressure, as in the Muzaffargarh Dispensary, where rooms at present used as store-rooms could easily, if needful, be utilised for patients with comparatively little trouble. During the year 1898 the total number of operations performed in the Mayo Hospital, Lahore, only amounted to 3,523, which is an increase of 268 on the previous year, the operations for cataract being the largest item. It is satisfactory to learn from the Report of the Inspector-General of Civil Hospitals that very considerable improvement has taken place in the manner in which diseases of the eye are treated, and that some of the assistant surgeons have now attained a high degree of excellence in operating for cataract; also that operations for stone have risen in number, and have been attended with a large measure of success. Cutting for stone in a great many of the hospitals is now practically discarded, except in a few cases where crushing is not possible. Unfortunately the instruments are very costly, and they need to be of different sizes for different ages, so necessitating a large number the purchase of which involves an outlay which many of the dispensaries cannot afford. But more and more instruments are brought each year, and very few natives willingly undergo the cutting operation, the great majority of the people knowing all about the crushing operation and appreciating it thoroughly. Ovariotomy was performed at the Lady Aitchison Hospital, Lahore, five times during the year by Dr. Elizabeth Bielby, and resulted in only one death. The number of cases of natural labour treated at the hospitals, or at the women's homes, by *dáis* and lady doctors has largely increased; and as more female medical aid is available, and native women come to understand that real help can be had in their time of trouble, the applications are bound to increase, till instead of being counted by the hundred they will be reckoned by the thousand. The total of persons vaccinated in 1898, according to the vaccinator's figures, was 42,000, with the result that there were only 67 cases of small-pox throughout the whole of the Punjab. This, coupled with the fact that there were only three cholera patients, is a quite unprecedented state of things.

